# Natural occurrence and production of tenuazonic acid in wine grapes in Argentina

**DOI:** 10.1002/fsn3.577

**Published:** 2018-02-23

**Authors:** Luciana P. Prendes, Ariel R. Fontana, María G. Merín, Agustina D´ Amario Fernández, Rubén Bottini, María L. Ramirez, Vilma I. Morata de Ambrosini

**Affiliations:** ^1^ Facultad de Ciencias Aplicadas a la Industria Universidad Nacional de Cuyo San Rafael Argentina; ^2^ Consejo Nacional de Investigaciones Científicas y Técnicas (CONICET) Sede Central Buenos Aires Argentina; ^3^ Instituto de Biología Agrícola de Mendoza Consejo Nacional de Investigaciones Científicas y Técnicas‐Universidad Nacional de Cuyo Chacras de Coria Argentina; ^4^ Departamento de Microbiología e Inmunología Facultad de Ciencias Exactas Físico‐Químicas y Naturales Universidad Nacional de Río Cuarto Río Cuarto Argentina

**Keywords:** *Alternaria*, environmental factors, tenuazonic acid, wine grapes

## Abstract

A survey was carried out to determine natural occurrence of tenuazonic acid (TA) in healthy and rotten wine grapes samples from different varieties (*n* = 37) collected during 2016 vintage in the region of DOC San Rafael (Argentina). In addition, inoculation experiments with three *Alternaria alternata* strains in wine grapes were done to elucidate TA production and its major influencing factors. The 16.2% (6/37) of total wine grape samples showed TA contamination with 4% (1/25) of incidence in healthy samples (77 μg·kg^−1^) and 42% (5/12) in rotten samples (10–778 μg·kg^−1^). Malbec, Cabernet Sauvignon, and Syrah varieties showed TA contamination, whereas Bonarda, Ancelota, Torrontés, Semillón, and Chenin did not. During inoculation experiments in wine grapes, two of three strains were able to produce TA among the evaluated conditions and the highest TA production was observed at 15°C and 25°C after 24 days of incubation. Nutritional composition of grapes results appropriate for *A. alternata* infection and TA production and, together with the adequate field conditions, favors TA natural occurrence in wine grapes.

## 1 INTRODUCTION

Tenuazonic acid (TA) is one of the major mycotoxin produced by some *Alternaria* species (Alexander et al., 2011; Arcella, Eskola, & Gómez Ruiz, 2016). TA has shown a high toxicity and it has been associated to precancerous changes in the esophageal mucosa of mice fed with it at 25 mg·kg^−1^ body weight per day during 10 months. It has also been linked to a human hematological disorder, “onylai,” a form of thrombocytopenia (Scott, 2004; Viñas, Bonet, & Sanchis, 1992; Yekeler, Bitmiş, Ozçelik, Doymaz, & Çalta, 2001). TA presence has been reported in different food commodities such as tomatoes, cereals, and their derivative products as well as fruit juices, beer, wine, edible oils, spices, and infant food among the most important ones (Asam, Lichtenegger, Liu, & Rychlik, 2012; Hickert, Krug, Cramer, & Humpf, 2015; López et al., 2016; Prelle, Spadaro, Garibaldi, & Gullino, 2013; Siegel, Rasenko, Koch, & Nehls, 2009; Zhao, Shao, Yang, & Li, 2015). Most worrisome, it has recently been detected in human urine confirming its frequent consumption (Asam, Habler, & Rychlik, 2013; Hoevelmann, Hickert, Cramer, & Humpf, 2016).

In October 2011, the EFSA (European Food Safety Authority) panel on contaminants in the Food Chain (CONTAM Panel) published its first risks assessment on *Alternaria* toxins in food and feed (Alexander et al., 2011). This work suggested that *Alternaria* toxins are of high concern for public health but results were not conclusive encouraging more studies to assess the real extent of food contamination. In 2013, five leading compounds among *Alternaria* toxins (TA, alternariol, alternariol monomethyl ether, altenuene, and tentoxin) were subjected to the current mandate 520/2013 of the European Commission to CEN for development of a standardized analytical method (European Committee for Standardization, 2013). Recently, EFSA published a dietary exposure assessment to *Alternaria* toxins in European commodities for European population (Arcella et al., 2016). In this second assessment the dietary exposure to TA was the highest among the different *Alternaria* toxins and higher than that of the 2011 report (Alexander et al., 2011). Such difference was linked to the higher availability of occurrence data (better food coverage) and the higher levels reported for some food commodities, major contributors to the exposure. Nevertheless, its conclusions encourage researchers to generate more data on the presence of *Alternaria* toxins in the relevant food products (e.g., fruit and fruit products, tomatoes and tomato‐based products, cereal‐based food for infants and young children, among others) in Europe as well as worldwide.

Wine is the result of complex interactions between fungi, yeasts and bacteria that commence in the vineyard and continue throughout the fermentation process until packaging (Fleet, 2003). Due to the increase in wine consumers’ awareness and attention to health risks related to food safety, monitoring TA occurrence to assess the extent of mycotoxin contamination in wine grapes results a major concern worldwide. Recently, natural occurrence of TA in Malbec wine grapes from DOC San Rafael (Argentina) has been reported (Fontana, Prendes, Morata, & Bottini, 2016). However, a wider survey with healthy and rotten wine grapes from different varieties could contribute to establish a more accurate extent of TA contamination.

*Alternaria* spp. is part of the main wine grape mycobiota from different winemaking regions worldwide (Kakalíková, Jankura, & Šrobárová, 2009; Rousseaux, Diguta, Radoï‐Matei, Alexandre, & Guilloux‐Bénatier, 2014; Steel, Blackman, & Schmidtke, 2013). In particular, *A*. *alternata* has been reported as the main component of Malbec wine grape mycobiota from DOC San Rafael (Argentina). Also, a high frequency and levels of TA production in ground rice‐corn steep liquor medium by *A*. *alternata* strains have been described (Prendes, Merín, Andreoni, Ramirez, & Morata de Ambrosini, 2015). Moreover, we recently found high and sustained levels of TA production by three *A*. *alternata* strains previously isolated from Malbec in a synthetic nutrient media similar to grape composition under water activity (*a*_W_) and temperature conditions normally found during wine grape ripeness in the field (Prendes, Zachetti, Pereyra, Morata de Ambrosini, & Ramirez, 2017). However, since food components and nutrient availability may interfere in metabolite biosynthesis of contaminant fungi (Barkai‐Golan & Paster, 2008), elucidating TA production and its major influencing factors in wine grapes is necessary.

Therefore, the aims of this work were to evaluate the natural occurrence of TA in healthy and rotten wine grapes from different varieties at 2016 vintage and to study the effects of temperature and incubation time on TA production of *A*. *alternata* strains in wine grapes.

## 2 MATERIAL AND METHODS

### 2.1 Sampling procedure

During 2016 vintage, 25 healthy wine grape samples of *Vitis vinifera* L. cv. Malbec, Cabernet Sauvignon, Syrah, Bonarda, Ancelota, Torrontés, Semillón, and Chenin varieties from different vineyards distributed representatively in the region of DOC or DO (Denomination of Origin) San Rafael (Argentina) were sampled at harvest time. This wine grape‐growing region is located between 34.3° and 34.8° S latitude, 67.4° and 68.5° W longitude, and 500 and 800 m altitude. Briefly, each independent sample consisted of grape bunches collected at 1.5 m from the ground from 12 plants homogeneously distributed in a vineyard (a bunch per plant) to reach 1 kg approximately, following a procedure previously described (European Commission, 2006). In addition, 12 rotten wine grape samples from different varieties and vineyards were collected, which consisted of a grape bunch with symptoms of fungal infection each. A total of 37 samples were kept in plastic bags each and placed in ice‐cooled boxes during transportation to the laboratory. Then, each sample was completely ground (whole grape bunches) in a laboratory mixer and three replicate aliquots of each (2.5 g) were collected in 50 ml PTFE plastic tubes and stored at −20°C. Each replicate aliquot from a sample were submitted independently to the TA extraction procedure and subsequent detection and quantification of TA.

### 2.2 Tenuazonic acid extraction, detection, and quantification

TA extraction was done following a high‐throughput modified QuEChERS (quick, easy, cheap, effective, rugged, and safe) method previously developed for TA in wine grapes (Fontana et al., 2016). The final extract was resuspended in 0.5 ml mobile phase [(MeOH: 0.1 mol/L NaH_2_PO_4_ (2:1 v/v), adjusted to pH 3.2] and 20 μl was injected in the HPLC MWD system (DionexSoftron GmbH, Thermo Fisher Scientific Inc., Germering, Germany). The working wavelength for the analyte was 279 nm. HPLC separations were carried out in a Kinetex XB‐C_18_ column (4.6 mm × 150 mm, 5 μm) Phenomenex (Torrance, CA, USA) and TA mobile phase and running conditions were those described by Fontana et al. (2016). Samples were quantified using a matrix‐matched calibration. Limit of detection (LOD, signal‐to‐noise ratio 3) was 10 μg·kg^−1^ and the quantification limit (LOQ) (lowest concentration of the analyte with recovery within the range 70%–120% and relative standard deviation ≤20% by applying the complete analytical method) was 50 μg·kg^−1^.

Copper salt of TA (Sigma‐Aldrich, Steinheim, Germany) was converted into its free form as described in the literature (Siegel et al., 2009). Stock solutions of TA were prepared in methanol (MeOH). Further dilutions were prepared monthly in MeOH and stored in brown bottles at −20°C to ensure stability.

### 2.3 Fungal strains and inoculum preparation

Three *A*. *alternata* strains (5.5, 7.5, and 25.1) previously isolated and identified by morphological and molecular methods from asymptomatic Malbec wine grapes from DOC San Rafael wine grape‐growing region during 2011 and 2012 vintages were used (Prendes et al., 2015). They have also shown high but different production levels of TA in ground rice‐corn steep liquor medium and moderate to high pathogenicity grade in a detached berry test during a previous study (Prendes et al., 2015). In addition, these isolates have also shown production of TA in a synthetic media similar to grape composition under different environmental conditions (Prendes et al., 2017). Preparation of inoculum was done following a previously described technique with some modifications (Nally et al., 2013). Briefly, each *A*. *alternata* strain was placed separately on Potato‐Carrot‐Agar (PCA) medium Petri dishes and incubated at 20°C–25°C during 7–10 days under cool‐white fluorescent lamps with an alternating 8/16 light/dark cycle. After incubation, 4 ml of sterile water containing 0.05% (v/v) Tween 20 were poured into the dishes to remove the spores from the mycelium and the suspension was centrifuged at 10,000 g for 5 min at 4°C. The supernatant was discarded and the spore pellet resuspended in 1 ml of sterile 0.01% (v/v) Tween 20. The different fungal spore suspensions were prepared determining spore concentration in a Neubauer chamber and adjusting to 1.75 × 10^2^, 1.75 × 10^3^, 8.75 × 10^3^, 1.75 × 10^4^, 5 × 10^4^, 1.9 × 10^5^ spores·ml^−1^ by dilution for each strain tested.

### 2.4 Determination of minimum infective concentration of *A*. *alternata* in grapes

The minimum infective concentration (MIC) of each *A*. *alternata* strain was determined using a previously described phytopathogenicity assay with some modifications (Nally et al., 2013). Briefly, healthy detached berries from Malbec wine grapes (250 g·L^−1^ reducing sugar; 3.6 g·L^−1^ total acidity, expressed as tartaric acid; 180 mg·L^−1^ yeast assimilable nitrogen (YAN); pH 4.22) collected at 2015 vintage were surface disinfected with Na hypochlorite solution (1%, v/v) for 1 min, rinsed in sterile distilled water and dried at room temperature. A single wound (3 mm in diameter and 3 mm in depth) was made at the equator of each berry using the tip of a sterile dissecting needle. Twenty microliter of a spore suspension of an *A*. *alternata* strain (previously described) was poured into the wound. Treated grapes were sterile air‐dried and placed in Petri dishes (8 grape berries per dish) and incubated at 25°C and 100% RH during 5 days. At the end of the experiment, the incidence of grape rotting for each *A*. *alternata* spore suspension was calculated as follows: Incidence (%) = (number of decayed wounds/number of total wounds) × 100. Negative control consisted in grapes inoculated with 20 μl of sterile 0.01% Tween 20 (v/v). Each experiment used eight berries per replicate and three replicates per treatment in a randomized complete block design. The experiment was carried out twice. The lowest fungal spore suspension with an incidence of 100% was defined as MIC and determined for each *A*. *alternata* strain (5.5, 7.5, and 25.1).

### 2.5 Inoculation, incubation, and TA production in grapes

To evaluate the effect of temperature and incubation time (abiotic factors) on TA production in grapes by *A*. *alternata* strains 5.5, 7.5, and 25.1, the previous described assay was performed with some modifications. Twenty microliter of the MIC determined for an *A*. *alternata* strain (1.75 × 10^4^ spores·ml^−1^ for 5.5 and 25.1 and 5 × 10^4^ spores·ml^−1^ for 7.5)was poured into the wound of a disinfected grape berry. Treated grapes were sterile air‐dried and placed in Petri dishes (8 grape berries per dish) and incubated at three different temperatures (15°C, 25°C, and 35°C) and 100% RH during 10, 17, and 24 days. A full factorial design was used, where the factors were strain, temperature, and incubation time, and the response was TA production (total number of plates: 3 strains × 3 temperatures × 3 times of incubation × 3 replicates = 81 plates). The entire experiment was repeated twice.

To determine TA production, each plate (sample) was ground completely in a laboratory mixer and an aliquot of 2.5 g was collected in a 50 ml PTFE centrifuge tube to be stored at −20°C until submitted to mycotoxin extraction procedure according to the methodology described above.

### 2.6 Statistical analysis

TA concentrations were evaluated by analysis of variance (ANOVA) to determine the effect of temperatures, incubation times and *A*. *alternata* strains and two‐ and three‐way interactions. When the analysis was statistically significant, the post hoc Tukey's multiple comparison procedure was used. Statistical significance was judged at the level *p* ≤ .001. Statistical analysis was done using Statistica 7.0 for Windows Version 7.0. Surface response and contour map graph were produced using Statistica and Sigma Plot v.13.0 (Systat Software Inc., Hounslow, London, UK).

## 3 RESULTS AND DISCUSSION

The present work reports the natural occurrence of TA in healthy and rotten wine grape samples from different varieties destined for winemaking industry during 2016 vintage in the wine grape‐growing region of DOC San Rafael (Argentina).

**Figure 1:**
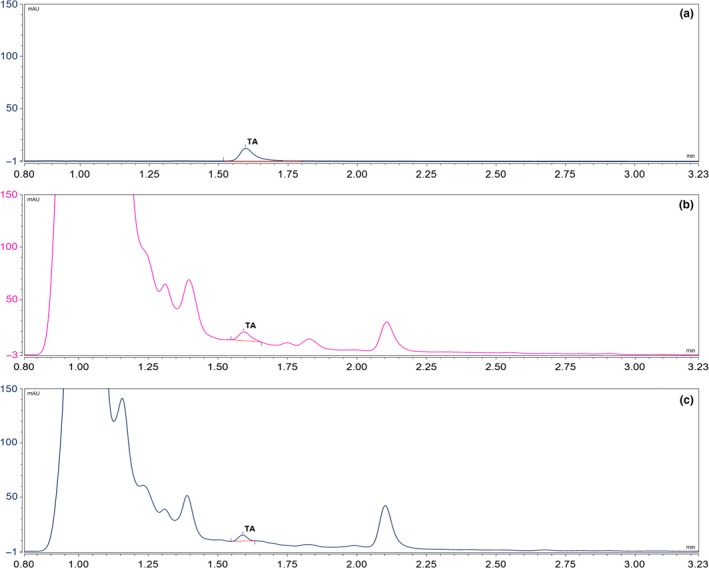
Extracted chromatograms at 279 nm. (a) Solvent TA standard at 1 mg·L^−1^, (b) rotten wine grape sample (Cabernet Sauvignon) with a TA level of 367 ± 40 μg·kg^−1^, (c) healthy wine grape (Malbec) sample with a TA value of 77 ± 12 μg·kg^−1^

The 16% of total samples (6/37) showed TA contamination with 4% of incidence in healthy samples (1/25), corresponding to a Malbec variety sample (77 μg·kg^−1^) (Table 1). Meanwhile, the 42% (5/12) of samples with symptoms analyzed showed TA contamination in a range from 10 (<LOQ) to 778 μg·kg^−1^. Malbec, Syrah, and Cabernet Sauvignon varieties showed TA contamination, whereas none of the other varieties (Bonarda, Ancelota, Torrontés, Semillón, Chenin) did. Figure 1 shows the chromatograms corresponding to the extract of a rotten wine grape sample (Cabernet Sauvignon) and of a healthy wine grape sample (Malbec), together with the solvent TA standard.

**Table 1:** Tenuazonic acid occurrence in wine grapes during 2016 vintage at DOC San Rafael (Argentina)

Wine grape variety	Phytosanitary status	Number of positive samples^a^	TA (μg·kg^−1^)
Malbec	Healthy	1/10	77 ± 12
	Rotten	1/4	367 ± 40
Cabernet Sauvignon	Healthy	0/4	
	Rotten	2/2	104 ± 18; 778 ± 15
Syrah	Healthy	0/5	
	Rotten	1/3	<LOQ^b^
Malbec and Syrah	Rotten	1/1	133 ± 20
Bonarda	Healthy	0/2	
Ancelota	Healthy	0/1	
Torrontés	Healthy	0/2	
	Rotten	0/1	
Semillón	Healthy	0/1	
Chenin	Rotten	0/1	

a Number of samples with TA values over the LOD versus total samples. LOD (Limit of detection): 10 μg·kg^−1^.

b LOQ (Limit of quantification): 50 μg·kg^−1^.

The possibly higher incidence and TA levels present in rotten wine grapes could be a consequence of a better environment for *Alternaria* infection and mycotoxin production.

*Alternaria* is an opportunistic pathogen, whose predominance in DOC San Rafael and its ability to produce high TA levels have been demonstrated (Prendes et al., 2015, 2017). This study also shows that mycotoxins could be present even in healthy wine grapes, probably as a result of a previous infection in surrounding tissues or the capability of fungi to produce mycotoxins under unfavorable conditions for growth (Barkai‐Golan & Paster, 2008).

Comparing this study with our previous study carried out during 2015 vintage, there are differences between 2015 and 2016 vintages, concerning TA production in Malbec variety (Fontana et al., 2016). TA occurrence was higher during the first harvest season (2015), with an incidence of 57.1% (8/14). Nevertheless, during 2016 harvest season, we found a TA incidence of 14.3% (2/14). Such differences could be correlated with the higher levels of rainfall present in the last 2 months before sampling during 2015 vintage (Table 2). Rainfall increases the water activity (*a*_W_) in the grape surface at or near harvest, favoring fungal development as well as mycotoxin production, as already reported for several crops (Paterson & Lima, 2010; Rousseaux & Donèche, 2001). On the other hand, TA levels seem to be in the same order among healthy (10–96 μg·kg^−1^ in 2015; 77 μg·kg^−1^ in 2016) and rotten (595 μg·kg^−1^ in 2015; 367 μg·kg^−1^ in 2016) Malbec samples, regardless the vintage considered.

**Table 2:** Meteorological data 3 months before each harvest season in 2015 and 2016 at DOC San Rafael^a^

Year	Month	*T*_max_ (°C)	*T*_mean_ (°C)	T_min_ (°C)	RH (%)	Rainfall (mm)
2015	January	35.0	26.0	16.8	59	0.0
	February	30.1	22.1	14.6	61	6.3
	March	29.5	21.4	14.0	65	10.3
2016	January	30.8	22.8	16.3	69	1.3
	February	31.8	23.3	15.4	63	0.0
	March	27.9	19.3	12.1	69	0.0

*T*_max_, maximum temperature; *T*_mean_, mean temperature; *T*_min_, minimum temperature; RH, relative humidity.

a INTA, Instituto Nacional de Tecnología Agropecuaria‐ EEA Rama Caída (DOC San Rafael).

TA occurrence has been described in several vegetables and fruits including tomatoes, tangerines, melons, peppers, apples, raspberries, citrus fruits, carrots, and lentils, among others (Alexander et al., 2011; Arcella et al., 2016). In agreement with our work, the maximum levels of *Alternaria* mycotoxins reported in marketed products were in the range 1–10^3^ μg·kg^−1^; higher levels were found in samples visibly infected by *Alternaria* rot, that is in products not suitable for consumption (Ostry, 2008). Mikušová, Sulyok, and Šrobárová (2014) have found TA natural occurrence in dried wine grape berries from three Slovak winemaking regions, with concentrations ranging from 700 to 31,000 μg·kg^−1^, being the lower value similar to that observed in one of our rotten sample (778 μg·kg^−1^) (Table 1). Recently, López et al. (2016) analyzed the natural occurrence of *Alternaria* mycotoxins (AOH, AME, TA, ALT, and TEN) on diverse commodities and TA was found in 27% of the samples. The highest concentrations were found in cereals, tomato sauces, figs, wine, and sunflower seeds, whereas the lowest ones were present in healthy fresh fruits, suitable for human consumption such as apples, olives, citric fruits, and tomatoes. TA levels found in fresh fruits suitable to human consumption (0–5 μg·kg^−1^) were significantly lower than the levels found in healthy wine grapes reported in the present work (77 μg·kg^−1^).

Fresh fruits would probably not contribute significantly to the human exposure to *Alternaria* toxins, because of the direct rejection of those visibly infected by their consumers (Alexander et al., 2011). However, wine grapes destined for vinification could represent a risk, due to their frequent utilization despite their phytosanitary status. In fact, natural occurrence of AOH, AME, and TA has already been reported in grape juice and wine (Broggi et al., 2013; Delgado & Gómez‐Cordovés, 1998; Fan, Cao, Liu, & Wang, 2016; Lau et al., 2003; López et al., 2016; Pizzutti et al., 2014; Scott, Lawrence, & Lau, 2006). In particular, wines from the Netherlands have shown TA levels in the range from 5 to 46 μg·kg^−1^. In that way, wine could significantly contribute to an increase in the ingestion of *Alternaria* mycotoxins in the human diet (Logrieco, Moretti, & Solfrizzo, 2009).

Fungal growth and mycotoxin production are the result of complex interactions between diverse biotic and abiotic factors, and knowledge of the effect of each involved factor is crucial for prediction, understanding, and prevention of mycotoxins contamination in food and by‐products (Sanchis & Magan, 2004). In a previous work, we have found that temperature, *a*_W_, incubation time and strain type, were influencing factors in TA production by three *A*. *alternata* strains isolated from wine grapes in a synthetic nutrient media similar to grape composition (SN media) (Prendes et al., 2017). However, several works have demonstrated that fungal strains, producers of toxins in synthetic media, were not producers in natural substrates (Betina, 1989; Viñas et al., 1992). To investigate these possibilities, this work analyzed for the first time TA production in wine grapes and the effect of temperature (15°C, 25°C, and 35°C) and incubation time (10, 17, and 24 days) using the three *A*. *alternata* strains that previously showed toxin production in a medium similar to grape composition (SN media) (Prendes et al., 2017).

The use of MIC for each evaluated *A*. *alternata* strain clearly ensures the infection of all inoculated grapes (100%) and allows a better comparison among strains, because they present differences in their MIC, 1.75 × 10^4^ spores·ml^−1^ for strains *A*. *alternata* 5.5 and 25.1 and 5 × 10^4^ spores·ml^−1^ for strain *A*. *alternata* 7.5. Importantly, MIC obtained for each *A*. *alternata* strain was generally in agreement with the grade of pathogenicity showed for the same strain in grapes (Prendes et al., 2015).

**Figure 2:**
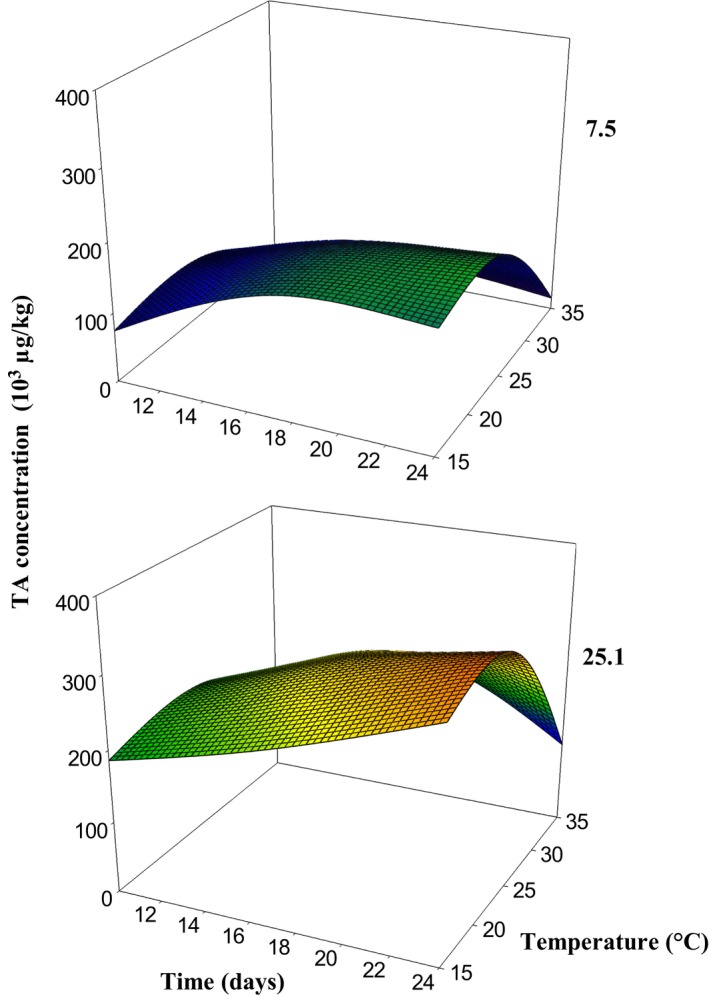
Tenuazonic acid levels (μg·kg^−1^) produced by strains of *Alternaria alternata* 7.5 and 25.1 in Malbec grapes at different temperatures and incubation times

During the study, strain 5.5 did not show TA production at any of the conditions evaluated (data not shown). Meanwhile, the strains 7.5 and 25.1 showed TA production at all temperatures and incubation times evaluated, with the second strain producing higher TA levels than the first one (Figure 2). The highest TA production was observed at 15 as well as 25°C for both strains after 24 days of incubation (175 × 10^3^ μg·kg^−1^ and 320 × 10^3^ μg·kg^−1^ for strains 7.5 and 25.1, respectively).

These results were partially in agreement with those obtained in SN media. The strain which produces the highest level of TA in the SN media also produces the highest level in grapes, whereas the *A*. *alternata* strain with the lowest TA production level in SN media was not able to produce it in grapes at any of the assayed conditions. Optimum conditions for TA production between the two substrates were also different. Maximum TA production was reached only at 25°C (not at 15°C) at 0.99 *a*_W_ in SN media, a condition probably similar to that of the inoculated wine grapes (100% RH). In general, optimum incubation times were shorter in SN media (14 and 21 days) and TA levels were higher than those in grapes, possibly as a consequence of higher availability of the nutrients in the SN media. Nevertheless, the present findings suggest that wine grapes are an appropriate substrate for *Alternaria* growth and mycotoxin production. Also, temperature, incubation time, and strain type were influencing factors on TA production in this substrate as well as they were in the SN media.

**Figure 3:**
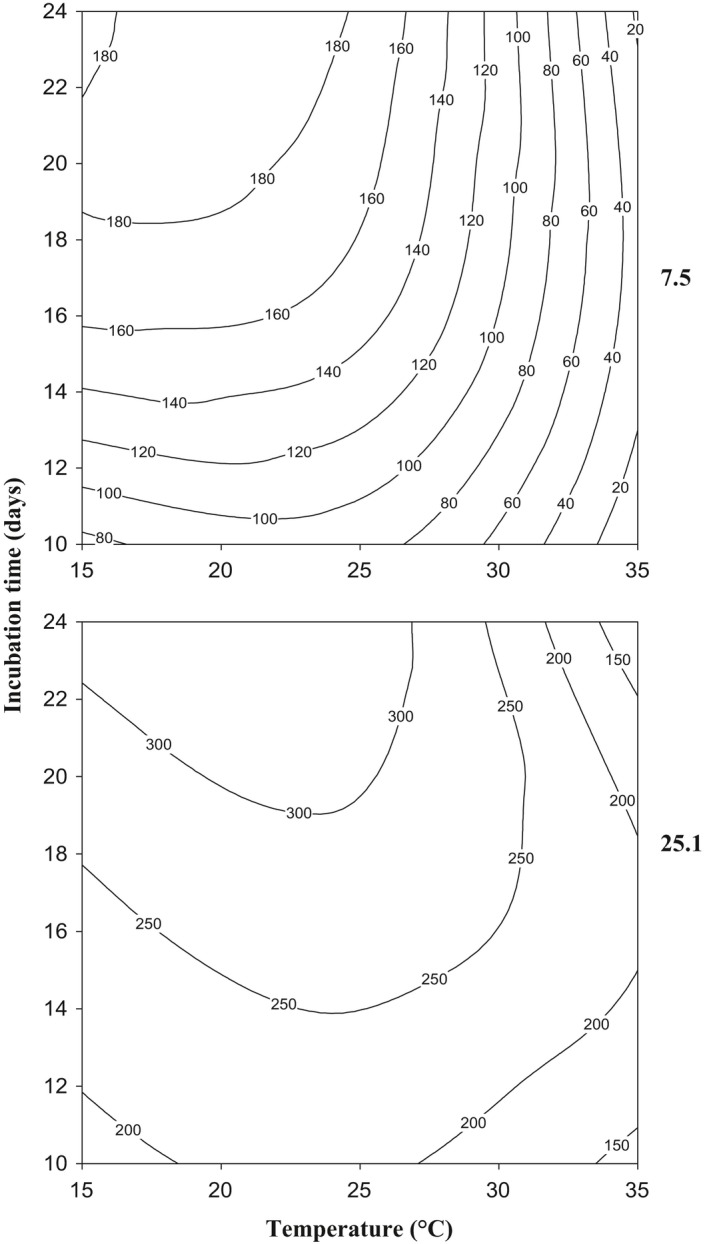
Two dimensional contour maps of tenuazonic acid production profiles of *A. alternata* strains 7.5 and 25.1 in relation to temperature and incubation time. The numbers of the isopleths refer to similar toxin concentrations (10^3^ μg·kg^−1^)

Data obtained were used to develop contour maps to identify the optimum conditions of temperature and incubation time and the range of conditions for production of different quantities of TA (Figure 3). As could be observed, strain 25.1 showed the widest spectrum of temperature and incubation time for TA production. Also, the elliptical shape of contour plots in factorial analysis indicates significant and prominent interactions between temperature and incubation time (Moyo, Gashe, Collison, & Mpuchane, 2003). This means that the effect of temperature on TA production depends on time of incubation and vice versa.

In order to determinate the effect of the variables evaluated on TA production by the two strains, an analysis of variance on the effect of single factor (strain, temperature, and incubation time) and the two‐ and three‐way interactions was carried out (Table 3). The *p*‐value of the model was <.0001, indicating that it was highly significant. The high *R* ^2^ value (*R* ^2^ = 99.45% and adjusted *R* ^2^ = 98.93%) indicated that almost 100% of the total variation is explained by the model, thus confirming the goodness of fit and the validity of it. All the single factors and their interactions, excepting interactions between strain and time and between strain and temperature, were statistically significant (*p* < .001; Table 3) on TA production by both strains evaluated, being the strain factor the most important one (*F*‐ Snedecor: 1471.574; Table 3).

**Table 3:** Analysis of variance on the effects of different strains (*S*), temperatures (*T*), and incubation times (*i*) and their interactions on tenuazonic acid production by two *Alternaria alternata* strains in wine grapes

Source of variation	Sum of squares	Degrees of freedom	Mean square	*F*‐ Snedecor
*S*	140700.6	1	140700.6	1471.574^a^
*T*	106783.4	2	53391.7	558.419^a^
*i*	37656.8	2	18828.4	196.925^a^
*S* × *T*	1249.2	2	624.6	6.533
*S* × *i*	82.1	2	41.0	0.429
*T* × *i*	19595.3	4	4898.8	51.236^a^
*S* × *T* × *i*	5937.3	4	1484.3	15.524^a^

*R* ^2^ = 99.45%; *R* ^2^ (adj) = 98.93%.

a *p *<* *.001.

Our findings represent important evidence that fruit can present *Alternaria* mycotoxins contamination when the appropriate conditions for fungal growth and/or mycotoxin production are given, as previously observed (Barkai‐Golan & Paster, 2008). In addition, the knowledge of the interactions among the different temperatures, *a*_W_ and incubation times performed in the present work and in a previous one (Prendes et al., 2017) provides useful information to predict the possible risks of *Alternaria* toxins contamination during maturation of wine grapes in the field until the start of vinification. TA production in wine grapes under a wide range of environmental conditions explains its natural occurrence reported in the present work.

## 4 CONCLUSION

This study reports TA occurrence in healthy and rotten wine grapes belonging to different varieties at harvest from the wine grape‐growing region of DOC San Rafael (Argentina). TA occurrence and its levels seem higher in rotten wine samples and only Malbec, Cabernet Sauvignon, and Syrah varieties showed TA contamination. It also reports that wine grapes are an adequate substrate for TA production and its highest value was observed at 15°C and 25°C after 24 days of incubation during inoculation experiments with *A*. *alternata* strains. Altogether these results show that field conditions that promote *A*. *alternata* growth and/or TA production also favor TA natural occurrence in wine grapes.

This study also contributes to evaluate the extent of grapes contamination with TA as well as to establish concentration limits for this toxin since there is a need to generate more data on the presence of *Alternaria* toxins in relevant food commodities (Arcella et al., 2016). A future analysis of the five leading compounds among *Alternaria* toxins (TA, AOH, AME, ALT, and tentoxin) in wine grapes as well as during the vinification process will contribute to understand the real exposure of wine consumers to *Alternaria* mycotoxins worldwide. Finally, with *Alternaria* presence as a toxicological risk, steps must be taken to control it as well as its toxin production in wine grapes.
